# Imaging of Light-Enhanced Extracellular Vesicle-Mediated Delivery of Oxaliplatin to Colorectal Cancer Cells via Laser Ablation, Inductively Coupled Plasma Mass Spectrometry

**DOI:** 10.3390/cells13010024

**Published:** 2023-12-21

**Authors:** Kara Chandler, Josh Millar, George Ward, Christopher Boyall, Tom White, Joseph David Ready, Rawan Maani, Keith Chapple, Robert Tempest, Joseph Brealey, Catherine Duckett, Sarah Haywood-Small, Simon Turega, Nick Peake

**Affiliations:** 1 Biomolecular Sciences Research Centre, Sheffield Hallam University, Howard Street, Sheffield S1 1WB, UK; 2 PerkinElmer AES (UK) Ltd., Chalfont Road, Seer Green, Beaconsfield HP9 2FX, UK; 3 Department of General Surgery, Sheffield Teaching Hospitals, NHS Foundation Trust, Sheffield S5 7AU, UK; 4 NanoFCM Co., Ltd., Medicity, D6 Thane Road, Nottingham NG60 6BH, UK

**Keywords:** laser-ablation inductively coupled plasma mass spectrometry, extracellular vesicle, oxaliplatin, photosensitizers, colorectal cancer

## Abstract

Extracellular vesicles (EVs) are lipid bilayer structures released by all cells that mediate cell-to-cell communication via the transfer of bioactive cargo. Because of the natural origin of EVs, their efficient uptake by recipient cells, capacity to stabilize and transport biomolecules and their potential for cell/tissue targeting and preferential uptake by cancer cells, they have enormous potential for bioengineering into improved and targeted drug delivery systems. In this work, we investigated the use of laser ablation inductively coupled plasma mass spectrometry (LA-ICP-MS) as a tool to measure the loading of platinum-based chemotherapeutic agents. The EV loading of oxaliplatin via co-incubation was demonstrated, and LA-ICP-MS imaging showed greater efficiency of delivery to colorectal cancer cells compared to free oxaliplatin, leading to enhanced cytotoxic effect. Further, the impact of EV co-loading with a porphyrin (C5SHU, known as ‘C5’) photosensitizer on oxaliplatin delivery was assessed. Fluorescence analysis using nano-flow cytometry showed dose-dependent EV loading as well as a trend towards the loading of larger particles. Exposure of OXA-C5-EV-treated colorectal cancer cells to light indicated that delivery was enhanced by both light exposure and porphyrins, with a synergistic effect on cell viability observed between oxaliplatin, EVs and light exposure after the delivery of the co-loaded EVs. In summary, this work demonstrates the utility of LA-ICP-MS and mass spectrometry imaging in assessing the loading efficiency and cellular delivery of platinum-based therapeutics, which would also be suitable for agents containing other elements, confirms that EVs are more efficient at delivery compared to free drugs, and describes the use of light exposure in optimizing delivery and therapeutic effects of EV-mediated drug delivery both in combination and independently of porphyrin-based photosensitizers.

## 1. Introduction

Extracellular vesicles (EVs) are small, membrane-enclosed nanoparticles that are produced by all cells and found in all biofluids [1]. They contain bioactive cargo, which they can efficiently shuttle between cells, tissues and organs [1,2], and this cargo both reflects the conditions of the donating cell and, upon uptake by a recipient cell, can contribute to altered cellular function [3]. Through sequencing, lipidomic and proteomic analysis, there is growing interest in the use of EVs as biomarkers to support clinical applications, and several studies have identified specific cargos as promising candidates to support diagnosis in a range of pathological conditions [4,5]. Furthermore, since EVs are physiological, stable, biocompatible nanocarriers, they also have great promise for the stable formulation and targeted delivery of therapeutic agents if their selective uptake by target cells can be appropriately engineered and optimized [6,7].

Colorectal cancer (CRC) is the third and second most common cancer worldwide in terms of incidence and mortality, respectively [8,9]. Despite improvements in screening, up to half of patients with CRC are still diagnosed in the later stages of the disease (stage III–IV), where the prognosis is poor—less than 10% of patients with stage IV disease survive for 5 years [10,11]. Therefore, early diagnosis is critical; however, even with early diagnosis and treatment with curative intent, up to 40% of patients develop disease recurrence [12,13]—with the disease returning either at the primary site or as distant metastasis. Altogether, this indicates limitations in current treatment approaches for CRC, the significant therapeutic complications linked to the development of treatment resistance, and an urgent need to develop better treatment strategies for patients, particularly with advanced CRC.

First-line treatment of CRC is usually resectional surgery to remove the affected tissue, coupled with adjuvant or neoadjuvant chemotherapy [14,15]. Common chemotherapy agents used for CRC include capecitabine, fluorouracil, and oxaliplatin, either used alone or as combination therapy in regimes such as CAPOX or FOLFOX [14,15,16]. Recent promising studies demonstrate that loading EVs with these agents can provide an effective means to stabilize, concentrate and deliver them to cancer cells both in vitro and in vivo [17,18,19,20]. Several methods have been employed to optimize EV loading with therapeutic agents, with co-incubation, electroporation, transfection and sonication as examples of techniques showing some degree of efficacy in the few comparative studies performed to date [21,22,23]. The choice of “passive” methods (such as co-incubation) compared to “active” methods, including sonication and electroporation, needs to be a consideration depending on the polarity and size of the drug and will likely influence whether the cargo is internalised, incorporated or adsorbed onto the EV surface. Assessing loading and subsequent delivery requires analytical tools capable of the sensitive detection of the analyte under investigation, and fluorescent agents such as doxorubicin are readily detectable using fluorometry and fluorescence microscopy [24,25,26,27,28,29], but detecting non-fluorescent agents such as platinum-based drugs and analysis in tissues requires analytical equipment such as chromatography or mass spectrometry-based instrumentation [30,31,32,33,34,35,36].

In this study, we assessed the use of inductively coupled plasma mass spectrometry (ICP-MS) for monitoring EV loading and the delivery of oxaliplatin. This sensitive analytical technique can measure trace levels of elements, allowing the quantitative study of elements such as platinum (Pt^195^), which are not present at significant levels in physiological tissues. Furthermore, by coupling ICP-MS to laser ablation (LA-ICP-MS), elemental signatures can be spatially localized from a solid sample, which has been applied in previous studies to assess the distribution of platinum-based chemotherapeutics in CRC plasma, pre-clinical models and patient tissues [37,38,39,40]. We used ICP-MS to quantify the efficiency of drug loading into EVs, followed by LA-ICP-MS, to assess delivery into CRC cells in culture using multi-element detection to align Pt^195^ distribution with Mg^24^, allowing for the confirmation of cellular localization.

Finally, using this quantitative analytical platform, we assessed whether the use of photosensitizer agents could promote the release of oxaliplatin from EVs, allowing more efficient control of release rate, delivery and distribution and improving subsequent chemotoxicity. We have previously reported the synthesis and phototoxicity of novel porphyrin-based photosensitizers [41]. We therefore assessed the impact of co-loading porphyrins with oxaliplatin into EVs and subsequent photoexposure using LA-ICP-MS to assess oxaliplatin release and delivery and chemotoxicity assays to assess the impact of photosensitizer-linked EV-mediated oxaliplatin delivery for the targeting of CRC cells.

## 2. Materials and Methods

### 2.1. CRC Cell Culture

The human primary colorectal cancer cell line SW480 and the isogenic patient-matched metastatic line SW620 were obtained from the European Collection of Authenticated Cell Cultures (ECACC) and cultured routinely in high glucose DMEM + GlutaMAX + pyruvate (Gibco, Thermo-Fisher, Leicestershire, UK), 10% *v*/*v* fetal bovine serum (FBS; Gibco, Thermo-Fisher, UK) and 1% *v*/*v* penicillin/streptomycin (P/S; Lonza Ltd., Slough, UK). The cells were maintained in a humidified incubator at 37 °C and 5% CO_2_ in air and checked for mycoplasma with MycoAlert© Detection kit (Lonza Ltd., UK) every 6 months with negative results throughout. For EV isolation, SW480 cells were subcultured into WHEATON^®^ CELLine™ AD-1000 Bioreactor flasks (DWK Life Sciences, GmbH, Wertheim, Germany). Cells (2.5 × 10^7^) in 15 mL of DMEM + 10% FBS were added to the inner cell compartment, and 500 mL DMEM + 10% FBS into the outer media compartment. After 10 days equilibration, the media in the cell compartment was replaced with DMEM supplemented with 10% *v*/*v* of Gibco EV-depleted FBS (Thermo-Fisher, UK), and media in the outer compartment was replaced with DMEM + 5% normal FBS and 1% P/S. The media in the outer compartment was replaced weekly. The EV-enriched conditioning media (CM), harvested weekly, was centrifuged at 300× *g* for 5 min (21 °C). The supernatant was collected and centrifuged again at 2000× *g* for 5 min (21 °C). The supernatant was then used to isolate EVs on the same day or stored at −80 °C until further use.

### 2.2. EV Isolation and Characterization

EVs were isolated from media harvested from bioreactor cultures using size-exclusion chromatography (SEC). First, media was concentrated to a volume of 0.5 mL using Vivaspin^®^ 20 (100 kDa MWCO, Sartorius, Göttingen, Germany) at 3000 g, 4 °C. Econo-pac columns (Biorad, Watford, UK) were packed with 10 mL sepharose CL-2B (GE Healthcare, Uppsala, Sweden), and EVs from the concentrated media eluted in PBS. 20 0.5 mL fractions were collected for downstream analysis before pooling of EV-rich fractions and further EV characterization. Following MISEV guidelines [42], a series of EV characterization steps were undertaken. Protein concentration in SEC fractions was performed via BCA assay using Pierce™ bicinchoninic acid (BCA) assay kit (Thermo Fisher, Carlsbad, CA, USA) calibrated to a bovine serum albumin standard curve. Dissociation-enhanced lanthanide fluorescence immunoassay (DELFIA©) was used to assess the expression of EV surface markers CD9, CD63, and CD81 in SEC fractions and pooled samples. Briefly, 100 µL samples were incubated overnight at 4 °C in Nunc 96-well MaxSorp™ ELISA plates, then blocked with 1% *w*/*v* BSA in PBS for 2 h at RT before the addition of primary antibodies to CD9 (Abcam PLC, Cambridge, UK), CD63 and CD81 (both Bio-rad Laboratories Inc., Watford, UK) in PBS + 0.1% BSA in PBS and incubation overnight at 4 °C. Antigen detection was performed via incubation with biotinylated anti-mouse IgG secondary antibody (Perkin-Elmer Life Sciences, Buckinghamshire, UK) in 0.1% *w*/*v* BSA in PBS for 1 h at RT, then Eu-N1 streptavidin conjugate (Revvity, Pontyclun, UK) in assay buffer (50 mM Tris-HCl, 0.9% *v*/*w* NaCl, 0.2% *v*/*w* BCA, 0.1% *v*/*v* Tween-20, 20 µM pentetic acid) for 45 min at RT. Time-resolved fluorescence (TRF, ex/em 320/615 nm) was measured after 5 min in DELFIA© enhancement solution (Revvity, UK) with a Wallac Victor2™ 1420 multilabel counter. Transmission Electron Microscopy was performed on pooled EV preparations at the Electron Microscopy Facility, Faculty of Science, University of Sheffield. EVs were transferred through absorption onto carbon-coated copper grids for 1 min, quickly dried with filter paper, and washed twice with water before negative staining in 2% uranyl acetate. After drying for 10 min, grids were then stained in Uranyl Formate and visualized on an FEI Tecani G2 Spirit BioTwin (PennState, State College, PA, USA) TEM, with images recorded using a Gatan Orius 1000B CCD camera and Gatan DigitalMicrograph software 3.5 (Gatan, Pleasanton, CA, USA). Nano-flow cytometry was performed using a NanoAnalyzer instrument (NanoFCM Co., Ltd., Nottingham, UK). Particle count, size and fluorescence in the PC5-A channel were recorded.

### 2.3. Chemotherapeutic Loading of EVs

After the pooling of EV-rich fractions following characterization, drug loading was first compared between sonication and co-incubation methods (Supplementary Figure S1). Oxaliplatin (Merck KGaA, Darmstadt, Germany) was prepared at 100 mM stock solution in water, doxorubicin at 50 mg/mL in PBS, and C5/C6 porphyrins synthesized at 500 µg/mL, as previously described [20]. For co-incubation loading, EVs were incubated with drugs at appropriate concentrations for 1.5 h at 37 °C in a shaking incubator. For sonication, EVs were incubated with drugs and subjected to 3 rounds of sonication at 20 Hz amplitude for 30 s, with 90 s resting between each round and 1 h resting at 37 °C at the end of the protocol. For the quantitation of loading efficiency, EVs were pelleted via ultracentrifugation at 100,000× *g* for 1.5 h, and analytes measured in both EV after lysis in 10% Triton X-100 and EV-depleted supernatant to produce a % efficiency using the equation EV^analyte^/(EV^analyte^ + supernatent^analyte^) × 100. Doxorubicin and porphyrins were measured using fluorescence (ex 590/em 590 nm) and calibrated to a standard curve. Oxaliplatin was quantified using solution mode on the ICP-MS (NexIon 350×, PerkinElmer, Waltham, MA, USA), and calibrated to a standard curve generated from analytical grade Pt^195^ (Merck KGaA, Darmstadt, Germany).

### 2.4. Cell Treatment and Drug Delivery Analysis

For drug delivery studies, SW480 cells were plated onto 12 mm glass coverslips in 12-well plates pre-coated with poly-D-lysine at a density of 2.5 × 10^5^ cells per well. After adhering overnight, treatments were applied as either chemotherapy agents in native form or as EV-loaded conditions. After treatment for indicated times, the cells were fixed using 10% formalin for 10 min before washing with PBS three times and storage in PBS until ready for analysis. For analysis of oxaliplatin delivery, fixed cells were dried using tissue, then glued to a microscope slide using superglue before drying in a vacuum desiccator for 30 min. Laser ablation was carried out using an ImageBio266 system (Elemental Scientific Lasers, Bozeman, MT, USA) with a wavelength of 266 nm, coupled to the NexIon 350× ICP-MS. Images were created using a 5 µM spot size with an 800 Hz repetition rate and a scan speed of 16 µm/s and a laser fluence of 1.3 J/cm^2^. The acquisition of Mg^24^ and Pt^195^ was performed using a dwell time of 0.02 mL/ms and ICP RF of 1300 W. Bright-field microscopy images were also captured using a 20× objective for comparison. For analysis of porphyrins, fluorescent microscopy was used (ex 590/em 590 nm), with DAPI counterstain (Vectashield antifade mounting medium, Vector Laboratories Inc., Newark, NJ, USA). Exposure of EVs and cells to light was performed using 15 min exposure on a 15 W white light box; otherwise, cell and EV preparations were kept in foil to prevent light exposure.

### 2.5. Cytotoxicity Assays

SW480 cells were plated into 96-well plates in DMEM + 10% FCS at 5000 cells per well overnight. The following day, the cells were treated with appropriate concentrations of either chemotherapy agents in native form or with EV-loaded agents alongside vehicle-only control wells. Treatments were performed for 72 h, then the Alamar Blue assay was performed via the addition of Resazurin sodium solution at a final concentration of 0.03 mg/mL for 4 h, and fluorescence was measured (ex 530/em 590 nm). Crystal violet assay was performed on 96-well plates treated as above, and after the complete removal of culture media, the cells were fixed via the addition of 10% formalin solution for 10 min before the addition of 0.5% crystal violet solution for 30 min. The plates were then washed 3 times with water and crystal violet solubilized using 10% acetic acid, and then the absorbance was measured at 570 nm.

### 2.6. Statistical Analysis

The normality of distribution was first assessed using the Shapiro–Wilk test. Depending on the distribution of the data, comparisons between the 2 groups were assessed either using *t*-test (parametric) or Mann–Whitney test (non-parametric). For multiple comparisons, data were analyzed either using ANOVA with Bonferroni post hoc test (parametric) or Kruskal–Wallis with Dunn’s post hoc test (non-parametric). All analysis was performed in GraphPad Prism 8.1.1 (GraphPad Software Inc., Boston, MA, USA) and significance was accepted at *p* < 0.05.

## 3. Results

### 3.1. Isolation of EVs via SEC from SW480 Bioreactor Culture Produces EVs with Expected Size, Structure and Marker Profile

**Figure 1:**
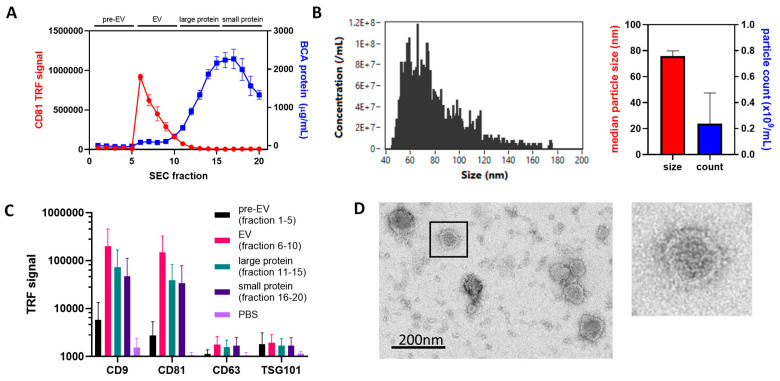
Extracellular vesicles isolated via size-exclusion chromatography. EVs were isolated from AD-1000 bioreactors via SEC with 20 0.5 mL fractions collected, with CD81-positive particles eluting in fractions 5–10 and later elution of other proteins ((**A**), n = 3). Pooling fractions 5–10 provided robust EV stocks with a median particle size of ~80 nm ((**B**), n = 3). Pools were positive for CD9, CD81 and CD63 using DELFIA ELISA ((**C**), n = 3), and the presence of double-membraned particles was confirmed using transmission electron microscopy (**D**).

To obtain high yields of purified EVs for the study, SW480 cells were cultured in bioreactors and conditioned medium harvested weekly. This EV-enriched conditioned medium was first concentrated using ultrafiltration and then separated using SEC. Analysis of the 20 fractions obtained via SEC showed a small spike in protein concentration between fractions 5 and 10 (Figure 1A), followed by the elution of most proteins in fractions 10–20, with a peak at fraction 17 (Figure 1A). Analysis of the same fractions using DELFIA ELISA for CD81 indicated that this EV surface marker was observed in fractions 6–10 (Figure 1A); it was therefore determined that fractions 1–5 were pre-EV elution, fractions 6–10 were pooled similarly to the EV-rich fractions, showing detectable proteins and the significant expression of CD81, and fractions 11–20 contained non-EV soluble proteins (indicated on Figure 1A). The pooled fractions 6–10 were then used as EV preparations for the study and were further characterized first by using nano-flow cytometry, showing a size distribution consistent with EVs [42,43], a mean (±SD) size of 75.8 (±4.1) nm, and preparations reaching 2.4 × 10^8^ particles/mL (Figure 1B). The isolated EVs showed detectable levels of CD9, CD81, CD63 and TSG101 via DELFIA ELISA (Figure 1C), and TEM confirmed the presence of lipid bilayer structures of appropriate size (Figure 1D). Overall, this characterization showed the successful isolation of high EV yields suitable for use in the study.

### 3.2. ICP-MS Shows Oxaliplatin Loading into EVs at Biologically Relevant Concentrations

**Figure 2:**
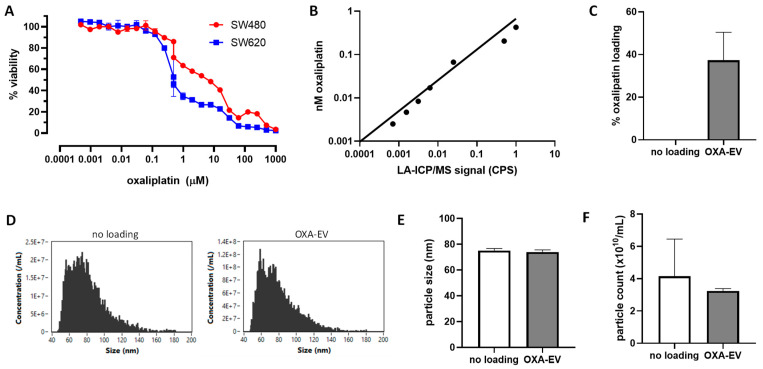
Sensitivity of SW cell model and ICP-MS measurement of oxaliplatin loading into EVs. To establish effective concentrations of oxaliplatin, dose–response curves were established on the isogenic colorectal cancer cell lines SW480 and SW620, with IC50 values between ~1 and 10 µM ((**A**), n = 3). Using a platinum standard, detection using ICP-MS was confirmed between 0.02 and 1 nM (**B**). The efficiency of oxaliplatin loading into EVs was then established using quantitation of post-loaded EVs (oxa-EVs) compared to EV-free supernatant ((**C**), n = 3). Oxa-EVs had a slightly altered size distribution compared to unloaded EVs (**D**), but there were no significant differences in overall median size (**E**), or total particle count (**F**) after oxaliplatin loading.

To begin assessing whether EVs could be used for oxaliplatin delivery, we first established concentrations of oxaliplatin suitable for testing in vitro. Using the CRC cell lines SW480 and SW620 in a viability assay based on Alamar Blue, we observed a cytotoxic range of oxaliplatin concentrations from 0.1 to 100 µM, with EC50 values of approximately 10 µM for SW480 and 0.5 µM for SW620 cells (Figure 2A). Next, we established a linear range for the detection of oxaliplatin via ICP-MS using a Pt^195^ standard, with detectable concentrations observed below 0.005 nM (Figure 2B). This enabled progression to load the isolated EVs with oxaliplatin. To select an effective loading method for water-soluble drugs, co-incubation was compared to sonication-mediated loading using an agent successfully loaded by other groups [23,24,25,26,27,28,29], a fluorescence assay and the fluorescent molecule doxorubicin were used, with co-incubation appearing more effective than sonication-mediated loading (Supplementary Figure S1A). Nano-flow cytometry indicated a tendency for larger EVs to be loaded (Supplementary Figure S1B,C). Once co-incubation was identified as the most effective loading method for hydrophilic drugs, oxaliplatin was then EV-loaded (oxa-EVs) using this method, with approximately 18.7% efficiency (Figure 2C) when EV-loaded drug was quantified via ICP-MS relative to the total amount of drug used to load. Nano-flow cytometry analysis of oxa-EVs indicated small changes to size distribution after loading with oxaliplatin (Figure 2D); however, there was no significant alteration in overall median size (Figure 2E) or particle counts (Figure 2E).

### 3.3. LA-ICP-MS Imaging Indicates Enhanced Delivery of Oxaliplatin into CRC Cells

**Figure 3:**
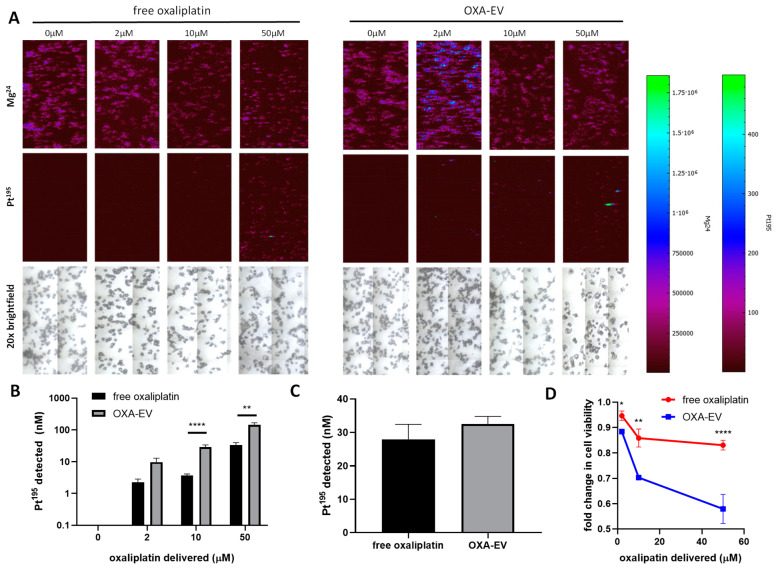
Oxaliplatin is delivered more effectively into cells using EVs. EV-loaded EVs were applied to SW480 cells using a range from 2 to 50 µM, corresponding to effective concentrations (Figure 2A). Using ICP-MS imaging, EVs showed more efficient delivery compared to free oxaliplatin ((**A**), showing Pt^195^ signal, with Mg^24^ and light microscopy imaging showing cells), with statistically significant increases at 3 h (**B**). By 24 h, uptake was saturated in both delivery modalities (**C**). This enhanced delivery results in a decrease in cell viability, as indicated by Alamar Blue assay (**D**). * = *p* < 0.05, ** = *p* < 0.01, **** = *p* < 0.0001.

After successfully loading oxaliplatin into EVs, we progressed to assessing the efficiency of delivery into a cell model. Using SW480 cells plated onto coverslips so that they could be used for imaging on the LA-ICP-MSI platform, SW480 cells adhered overnight were treated either with free oxaliplatin at effective concentrations of 2–50 µM to span the cytotoxic range for this cell line (Figure 2A). The detection of Mg^24^ and brightfield microscopy allowed the visualization of the treated cells, and Pt^195^ was detected as co-localizing with the Mg^24^ signal, indicating the uptake of oxaliplatin into the cells (Figure 3A, showing detection after 3 h of treatment). For the treatment with oxa-EVs, EV preparations were diluted and normalized to the loading efficiency of 18.66% (Figure 2C) before the treatment to deliver concentrations equivalent to the free oxaliplatin concentrations used. At 3 h post-delivery, the cellular accumulation of oxaliplatin after oxa-EV treatment was observed (Figure 3B) and quantified using CPS from the LA-ICP-MS calibrated to Pt^195^ (Figure 3B). At 10 µM and 50 µM concentrations, oxa-EV delivery resulted in statistically significantly greater cellular accumulation of oxaliplatin compared to free oxaliplatin (Figure 3B, *p* < 0.0001 and *p* < 0.01, respectively). This was dependent on the dose of oxaliplatin delivered; the cellular accumulation of Pt^195^ at 3 h increased from 2.25 nM to 9.7 nM with the 2 µM oxaliplatin treatment, from 3.6 nM to 28.8 nM with the 10 µM oxaliplatin treatment, and from 33.1 to 143.6 nM with the 50 µM oxaliplatin treatment. By 24 h post-delivery, significant differences between free oxaliplatin and oxa-EV were not seen, with a plateau observed of around 30 nM Pt^195^ using 10 µM oxaliplatin (Figure 3C, and Supplementary Figure S2). To assess whether this enhanced, rapid uptake of oxaliplatin had an impact on cytotoxicity, Alamar Blue assays were performed, indicating that oxa-EV increased cytotoxicity compared to free oxaliplatin, with 3%, 14% and 21% decreases in cell viability observed at 2 µM, 10 µM and 50 µM concentrations, respectively (Figure 3D, *p* < 0.05, *p* < 0.01 and *p* < 0.0001, respectively).

### 3.4. Loading of Porphyrins into EVs Is Concentration-Dependent and Biases towards Larger EVs

After demonstrating the successful delivery of oxaliplatin using oxa-EVs, we next investigated the use of the LA-ICP-MSI platform and Pt^195^ measurement to test whether oxaliplatin release, delivery and/or efficacy could be enhanced using photosensitizer (PS) agents. Since PSs such as porphyrins enable the conversion of light into reactive oxygen species, we tested whether this energetic transfer may affect EV membrane integrity, either before or after delivery into CRC cells. The PSs used in the study are third-generation bioconjugates between a tetra-phenyl porphyrin ring and four truncated fatty acids connected by a 3-phenyl ether linkage. The two PSs, 5CSHU and 6CSHU, differ in truncated fatty acid chain length by a CH_2_ extension (Supplementary Figure S3A). This single-chain length extension results in structural differences of hydrophobic surface area of 75 A^2^ and a total of eight nonpolar, potentially hydrophobic surface site interaction points [44]. This extension can be thought to provide potential extra hydrophobic interaction of 20 kJ mol^−1^ with the potential to interact with EVs differently.

**Figure 4:**
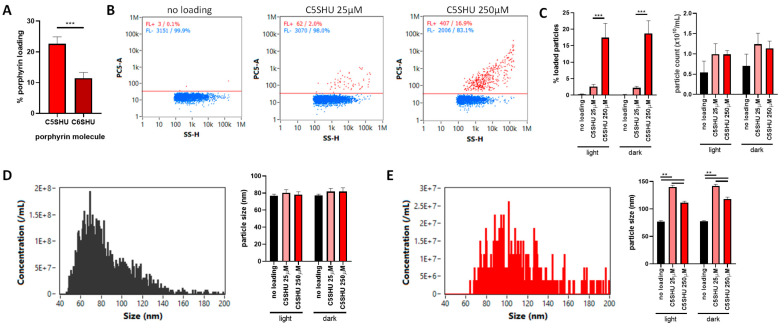
Porphyrin loading into EVs does not impact EV viability. The loading of porphyrins C5SHU and C6SHU into EVs was assessed using colorimetric analysis of EVs and EV-free supernatant at 420 nm, with the loading efficiency of C5SHU being significantly higher (**A**). Moving forward with C5SHU, loading efficiency was matched to concentration, with 2% EVs positive after loading at 25 µM, and 17% after loading at 250 µM (**B**). Exposure to light (15 min) did not significantly alter particle size or counts (**C**); however, notably, the size profile of total EVs (**D**) was different from the loaded EVs (**E**), with loading observed in larger EVs (110–140 nm, (**E**)). ** = *p* < 0.01, *** = *p* < 0.001.

To gain an understanding and allow comparison of PS efficacy for the C5SHU and C6SHU PS, the quantum yields for fluorescence and singlet oxygen formation were measured in DMF, an appropriate polar–organic solvent (Supplementary Methods S1 and S2, respectively). The fluorescence quantum yields provide a measure of how many of the absorbed photons are re-admitted as fluorescence was low for both PS at a value of 0.13–0.12, respectively (Supplementary Figure S3B). The single oxygen quantum yield was measured by trapping singlet oxygen formed using the molecule as a singlet oxygen trap, providing a measure of how many of the absorbed photons result in energy being re-emitted through the conversion of triplet oxygen to reactive singlet oxygen. The singlet oxygen quantum yield of both PS C5SHU and C6SHU gave similar values of 0.71 and 0.79, respectively (Supplementary Figure S3B), which represents a good conversion of absorbed light to singlet oxygen. The singlet oxygen and fluorescence quantum yields for the two PS are similar, as is expected for the comparison of these two PS; the porphyrin core in both molecules that controls the photochemistry of these molecules is identical with the PS, varying only in the chain length of the truncated fatty acid and not the electronic structure of the porphyrin ring. This means that while the photochemistry of the two molecules will remain the same, their ability to interact with environments non-covalently changes, so loading efficiency was assessed using fluorescence assay (Figure 4A). C5SHU was more efficiently EV-loaded compared to C6SHU (22.7% compared to 11.5%, *p* < 0.001) and was therefore used in subsequent experiments.

Because these PS molecules are fluorescent, we were able to characterize an individual EV level using nano-flow cytometry. This analysis indicated that loading concentration significantly affected loading efficiency in terms of the % of particles positive for C5SHU, with almost 5.8-fold more C5SHU-positive EVs after loading with 250 µM compared to 25 µM (Figure 4B,C, *p* < 0.0001). Pre-exposure to light did not immediately impact the amount of loaded C5SHU (Figure 4C), and neither C5SHU loading nor light exposure of loaded particles had a significant impact on the particle count (Figure 4C) or size (Figure 4D), indicating that no significant impacts were observed on EV viability or structure. Interestingly, when C5SHU-positive particles were compared to overall particles, loading appeared to bias towards larger EVs (Figure 4E)—a similar effect to the previous observations using doxorubicin (Supplementary Figure S1B).

### 3.5. Light Exposure and C5 Both Alter Oxa-EV Delivery

Due to its better loading profile, C5SHU PS was used to assess whether the PSs could impact the delivery of oxaliplatin from EVs by co-loading C5SHU and oxaliplatin. Using the oxaliplatin loading efficiency of 18.7% to normalize treatments (Figure 2C), EVs were co-incubated with 187 µM oxaliplatin either alone or in combination with 25 µM or 250 µM C5SHU to develop oxa-C5S-EVs with effective concentrations close to the oxaliplatin EC50 of 10 µM (Figure 2A) and the bioactive concentration of C5SHU [41]. SW480 CRC cells were then incubated with oxa-C5SHU-EVs, and delivery was monitored using the LA-ICP-MS platform. Analysis using fluorescent microscopy showed the successful delivery of C5SHU (Supplementary Figure S4), with increased cellular accumulation using the higher C5SHU concentration of 250 µM (Supplementary Figure S5A). The release of oxaliplatin from oxa-C5-EVs following light exposure was assessed via ICP-MS, and although no statistically significant differences were observed, a trend towards increased release of oxaliplatin after light exposure was observed (Supplementary Figure S5B), suggesting small impacts on drug release promoted by C5SHU activation.

**Figure 5:**
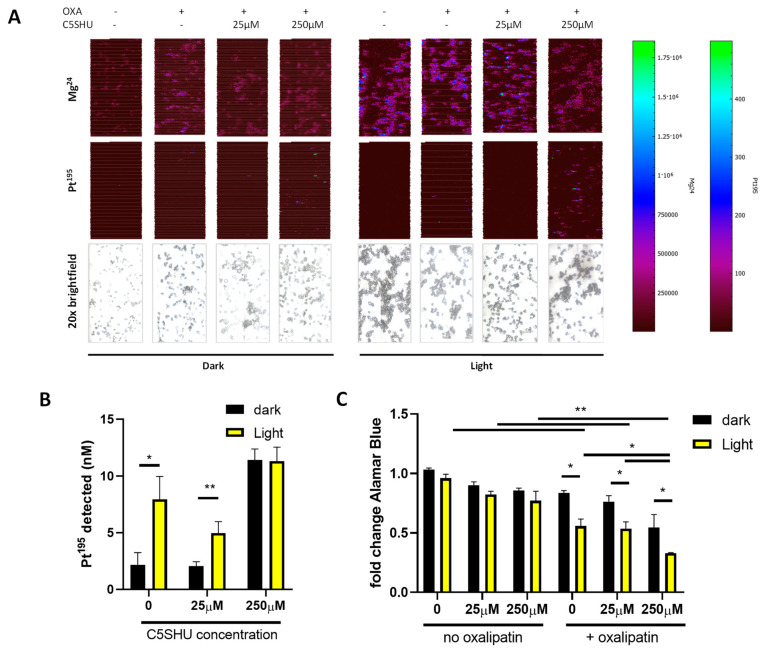
Light exposure and C5SHU porphyrin both impact on Oxa-EV delivery and effects. Loading into EVs does not impact EV viability. After co-loading of EVs with C5SHU, the impact of light exposure was assessed and showed a small but non-significant release of oxaliplatin from EVs (**A**). Significant increases in intracellular platinum were measured after EV delivery (3 h, followed by 24 h incubation, (**B**)). Co-loading with 250 µM C5SHU appeared to saturate the delivery of oxaliplatin (**B**). Both exposure to light and the presence of C5SHU caused significant loss of cell viability as measured via Alamar Blue assay (**C**) * = *p* < 0.05, ** = *p* < 0.01.

Next, LA-ICP-MSI was used to visualize the delivery of oxaliplatin to SW480 cells. The delivery of oxaliplatin was observed from oxa-C5-EVs (Figure 5A), with Pt^195^ co-localizing with Mg^24^, indicating cellular uptake. Quantifying Pt^195^ showed that 250 µM C5SHU loading had a significant impact on the delivery of oxaliplatin (Figure 5B, *p* < 0.0001) compared to 25 µM and no C5SHU (cellular detection of 11 nM ^195^Pt, compared to 5 nM and 8 nM, respectively). Interestingly, light exposure also had a significant effect, which was partially independent of C5SHU. Light alone (no C5SHU) significantly enhanced delivery from oxa-EVs (from cellular detection of 2 nM Pt^195^ to 8 nM, Figure 5B, *p* < 0.05), and oxa-EVs co-loaded with 250 µM C5SHU did not require light for significantly enhanced delivery (Figure 5B, ns). To confirm that this enhanced delivery also had an impact on cellular effects, Alamar Blue assays were performed. C5SHU in the absence of oxaliplatin had no significant effect on cellular viability (Figure 5C). Oxa-EVs significantly increased cytotoxicity as before (Figure 5C, *p* < 0.01), and interesting light exposure alone (no C5SHU) significantly enhanced the effect of oxaliplatin (Figure 5C, *p* < 0.05). Co-loading with 25 µM C5SHU did not significantly influence cell viability; however, 250 µM did significantly enhance the effect of Oxa-EV (Figure 5C, *p* < 0.05), and the combination of Oxa-C5-EV (250 µM) with light exposure was additive, showing the greatest decrease in cell viability (Figure 5C, *p* < 0.05). This additive effect was further validated with crystal violet assay, which showed that Oxa-C5-EV (250 µM) significantly reduced viability following light exposure (Supplementary Figure S5C, *p* < 0.05).

## 4. Discussion

In this study, we explored the use of ICP-MS as an analytical tool to measure the loading, cell delivery, and release of chemotherapeutic agents containing rare elements using the signal of elements not generally observed physiologically. Using liquid mode analysis, the amount of chemotherapeutic agents, such as platinum-based compounds like oxaliplatin, carboplatin and cisplatin, can be measured in EVs, which can be easily separated from unbound drugs using standard EV isolation methods. Furthermore, coupling laser ablation to ICP-MS and operating in imaging mode allows for the spatial visualization and quantification of chemotherapeutic delivery to cancer cells, making this a versatile platform to study EVs as chemotherapy delivery tools from loading, release profile and delivery.

The EV system used for this study was autologous EVs taken from bioreactor cultures of SW480 CRC cells, which were loaded and then delivered to SW480 cell cultures. The EVs were characterized following guidance from the MISEV 2018 guidelines [42] after isolation via SEC. Profiling the SEC fractions using the EV marker CD81 and BCA showed good separation of EVs from other proteins, and EV pools used for the study showed the expression of EV markers (CD9, CD63, and CD81), expected size distribution, and upon imaging via TEM, we were able to identify membrane-enclosed structures consistent with the size profile obtained via nano-flow cytometry.

SW480 and SW620 cells were exposed to oxaliplatin before proceeding with the study to confirm their sensitivity to the drug, and EC50 values of 0.5–10µM with greater sensitivity of the metastatic SW620 line were approximately consistent with some previous studies [45], although there is variability reported in the literature with significantly higher resistance reported in other studies [46]. This enabled us to assess whether EVs could be loaded with oxaliplatin at concentrations with the potential to deliver bioactive doses. SW480 cells were used for subsequent drug delivery studies, as the cell line was less sensitive to oxaliplatin, to assess the potential for EVs to deliver functional payloads with effects greater than conventional drug delivery. Platinum agents have been loaded into EVs previously; however, their delivery into cell models is less characterized due to the need for analytical tools like LA-ICP-MS. Our initial data on the sensitivity of this platform for Pt^195^ established high sensitivity and capability in terms of monitoring loading and delivery in physiological systems.

We used passive co-incubation as the EV loading technique after confirming the data presented in previous studies evidencing that this is more effective than active techniques, such as sonication and electroporation, for the loading of small, hydrophilic drugs [21,22,23]. Whilst this may not be appropriate for all drugs since many anticancer agents are hydrophobic, this seemed to work for the aqueous solutions of hydrophilic agents used in this work, and no adverse impacts on EV particle counts or size were observed, suggesting that this is an inert loading method that retains the biological properties of EVs. Another consideration of the loading method is that different approaches may also influence whether drugs are incorporated/internalised into EVs (and presumably protected from degradation or possible off-target toxicity) or simply adsorbed onto the surface. The approach here does not have a resolution to discriminate this, but further work to assess this is necessary in combination with work to establish the uptake, delivery and EV release mechanism. Drug loading efficiencies, assessed using either fluorometric assays or ICP-MS, were observed to be around 20% for doxorubicin, oxaliplatin and porphyrins, which is consistent with the range of loading efficiencies described by other groups [21,22,23,24,25,26,27,28,29,30,31,32,33,34,35,36]. Interestingly, because we used porphyrin molecules, which are fluorescent in part of this study, we were able to use single-particle nano-flow cytometry to further characterize this, observing that the loading efficiency was a function of loading concentration but that this was linked to the number of particles loaded, rather than as may be expected, the amount of drug loading into each EV. More careful characterization work concerning the use of EVs as a chemotherapeutic delivery system is now underway in our team in order to optimize loading per EV, optimize the yield of loaded EVs, and more carefully control each EV preparation since variability in particle number and loading efficiency between preparations was noted and we did not perform extensive tests to optimize particle number, which will be needed to take this approach further.

Another interesting observation made using nano-flow cytometry is that when compared to the total EV population, positively loaded EVs were significantly larger particles. The significance of this is not clear but was observed with both fluorescent molecules tested (doxorubicin and C5SHU porphyrin). It has been noted previously that EV loading is heterogeneous [47], so considering whether some populations or subtypes of EVs may be more susceptible to chemotherapy loading and others not susceptible at all requires more work if this approach is to be standardized for clinical use.

Porphyrins have been successfully EV-loaded in previous studies [21], and this work confirms that delivery into cells is rapid and efficient. The delivery of both oxaliplatin assessed using LA-ICP-MSI, C5SHU porphyrin assessed via fluorescence microscopy, alongside brightfield microscopy and overlaying ICP-MS signal with the physiological signal Mg^24^ showed that drugs were detectable intracellularly within 3 h, and that by 24 h, uptake was generally saturated. Further, delivery using EVs delivered this payload more effectively within the 3 h time frame, consistent with previous work on platinum drugs, porphyrins and other drugs [21,22,23,24,25,26,27,28,29,30,31,32,33,34,35,36]. The ability of EVs to deliver drugs applied at similar concentrations to conventional delivery methods with greater, more rapid efficiency means they have great potential for clinical application. EVs used clinically could be autologous (patient-derived) and biocompatible to minimize issues with immunological reactions [7]. They are known to be readily taken up by cancer cells [6], and their biologically physiological constitution means they have well-characterized surface chemistry potentially tailorable to targeted, cancer-specific delivery [48]. All these advantages are the subject of intense current development work, and the LA-ICP-MS platform demonstrated here makes it a useful analytical addition to support this development.

Porphyrins and other PSs are known to be cytotoxic and are used for photodynamic therapy [41,49] as they have radical-producing activity outside of cells. We observed that C5SHU porphyrins did not influence the viability of EVs even after exposure to light, and more interestingly, did not significantly influence the release of chemotherapy agents from EVs after light exposure—although a trend towards increased release of co-loaded oxaliplatin was noted. Overall, this suggested that photosensitizing Evs maintains their viability and structure, with potentially small effects on membrane integrity leading to some drug release. Given the small impacts observed, more work is needed to clarify this. Perhaps the most interesting experimental finding in this work is that light and C5SHU porphyrin both had a significant and partially independent impact on the delivery and cytotoxicity of EV-mediated oxaliplatin. It is known that light exposure can have a heat-mediated additive effect on photodynamic therapy, which may be what is observed here [49]. Further work will be enormously beneficial to understand the potential synergy between EV delivery, photosensitizers and light exposure, and the possibility of improving delivery, anticancer responses, and potentially reducing drug resistance in CRC patients.

This study utilized oxaliplatin, which is a key front-line agent used for the treatment of CRC, usually in combination with other agents, such as fluorouracil in regimes such as FOLFOX, either as adjuvant or neoadjuvant therapy [14,15,16]. The LA-ICP-MS platform is ideal for monitoring the delivery and uptake of these kinds of agents since ^195^Pt is not generally observed in measurable levels physiologically—as a result, LA-ICP-MSI has been used previously to measure oxaliplatin delivery in CRC tissue samples [37,38,39,40], and could be adapted towards drugs with other rare elements such as fluorouracil, capecitibine, and novel drugs developed using other metals [50]. The use of labels containing rare elements would also make it feasible to co-measure EV and rare element chemotherapeutics, making this a highly adaptable platform for the study of EV-mediated chemotherapeutic delivery, which will be applicable in terms of basic science and the pre-clinical and clinical pipeline. Through the use of inhibitors of EV uptake and endocytic sorting, the potential of single-EV nano-flow cytometry, and with the improving resolution of LA-ICP-MS, the potential to trace drug delivery from EV loading to cellular fate has the potential to provide valuable insights into the rapidly expanding field of EV-based drug delivery.

## 5. Conclusions

This study demonstrates for the first time that ICP-MS has great potential for the measurement of the EV loading, delivery and chemotoxic effects of chemotherapeutics containing rare elements such as platinum. Further, using porphyrin co-loading, we have demonstrated that the chemotherapy loading of EVs appears to bias towards larger particles and that higher drug loading concentrations influence not just the amount of drug per particle but apparently the number of drug-positive EVs. Finally, we uncover an independent effect of light exposure and photosensitizing porphyrins on the delivery and cytotoxic impacts of chemotherapy-loaded EVs.

## Data Availability

Raw data files are stored on a secure server under institutional data retention policies. The corresponding author can provide fully anonymized raw data upon request.

## Supplementary Materials

The following supporting information can be downloaded at: https://www.mdpi.com/article/10.3390/cells13010024/s1.
