# Supplementary material for: Imaging of Light-Enhanced Extracellular Vesicle-Mediated Delivery of Oxaliplatin to Colorectal Cancer Cells via Laser Ablation, Inductively Coupled Plasma Mass Spectrometry
Source: Cells. 2023 Dec 21;13(1):24. doi: 10.3390/cells13010024 (PMC10778274; doi:10.3390/cells13010024)
Supplement: Supplementary file 1 [file cells-13-00024-s001.zip › Chandler et al 2023 - supplementary information_R2.pdf]

Imaging of Light-Enhanced Extracellular-Vesicle Mediated Delivery of Oxaliplatin to Colorectal Cancer Cells by Laser-Ablation, Inductively-Coupled Mass Spectrometry

Chandler K [1], Millar J [1], Ward G [1], Boyal C [1]., White T [1]., Ready J [1,2], Maani R [1], Chapple K [3], Tempest R [1,4], Brealey J [4], Duckett C [1], Haywood-Small S [1], Turega S [1], Peake N [1,*]

Supplementary information

Method S1: High-Content Fluorescence Quantum Yield Experiment

Stock solutions of **PS**'s were made up to 50 µM with DMF, including standard (TPP). These solutions were applied to a Hellma quartz microplate, using an Eppendorf multipipette E3X, and diluted to 37.5 µM, 25 µM, 12.25 µM, 6.25 µM, and 2.5 µM with DMF. Absorption and fluorescence intensity measurements were taken at room temperature. Porphyrins were excited at the beginning of the first Q-ban, 516 nm A graph of integrated fluorescence intensity vs absorbance (at 516 nm) was plotted for every PS, and the quantum yield was calculated by inputting the gradients of the resulting straight line:

$$\phi_f = \phi_{st}\left(\frac{gradient_x}{gradient_{st}}\right) \quad (1)$$

Where $gradient_x$ is the slope of the unknown PS, $gradient_{st}$ is the slope of the standard (TPP), ϕst is the FQY of the standard [S1].

Method S2: High-Content Singlet Oxygen Quantum Yield Experiment

1,3-Diphenylisobenzofuran, stock solution (1mM) was made up in DMF on the day of the experiment. Solutions of the PS's (50 µM) were made up in DMF. These solutions were applied on to a Hellma quartz microplate, using an Eppendorf multipipette E3X. Absorption measurements were recorded at the discrete wavelength 313 nm with a resolution of 1 nm at room temperature. The microplate was irradiated periodically whilst shielded from natural light, after each period absorbance measurements were obtained. Absorbance vs. time plots were generated from the absorption data, and initial rates were determined from the resulting straight line. The high content format allows the simplification of data analysis, the rates were plotted against their corresponding PS concentration, in which the gradient can be inputted:

$$\phi_\Delta = \phi_{st}\left(\frac{gradient_x}{gradient_{st}}\right) \quad (2)$$

Where $gradient_{x}$¬ is the slope of the unknown PS, $gradient_{st}$ is the slope of the known standard, and ϕst is the known SOQY of the standard [S2].

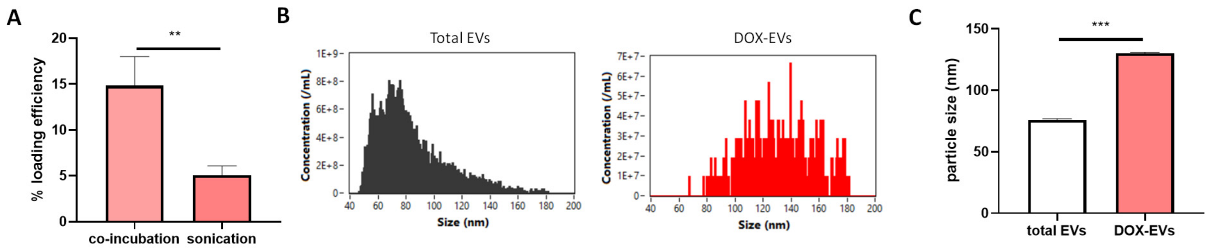

Supplementary Figure S1: Doxorubicin was used to validate EV loading methods, showing significantly better loading efficiency with co-incubation compared to sonication (A). EVs were assessed by nano-flow cytometry, and particles showing positive doxorubicin loading were larger compared to the total EV population (B, C).

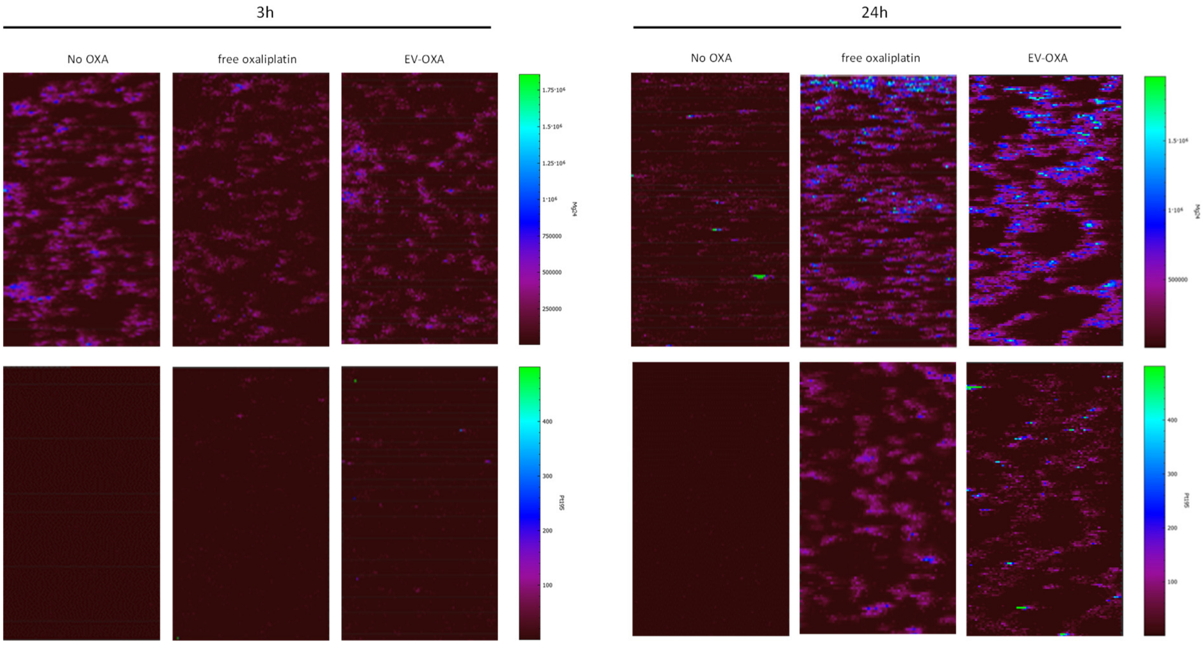

Supplementary Figure S2: The delivery of free oxaliplatin vs EV-OXA compared between 3h and 24, showing LA-ICP-MSI of Mg^{24} (top row) and Pt^{195} (bottom row) after treatment with 10µM oxaliplatin.

A

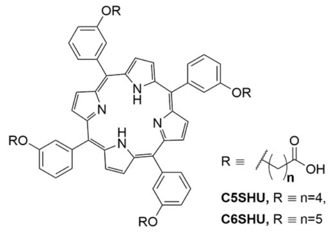

B

PS	Φ_f	Φ_Δ
C5SHU	0.13 ± 0.001	0.71 ± 0.02
C6SHU	0.12 ± 0.005	0.79 ± 0.02

Supplementary Figure S3: The porphyrin molecules C5SHU and C6SHU were used for EV loading and representative structures are shown (A). Fluorescence quantum yield (Φf) of PS in DMF and singlet oxygen quantum yield (ΦΔ) values for the PSs taken in DMF at room temperature are shown (B), values are represented in the form of mean ± σ. ** = $p < 0.01$, *** = $p < 0.001$.

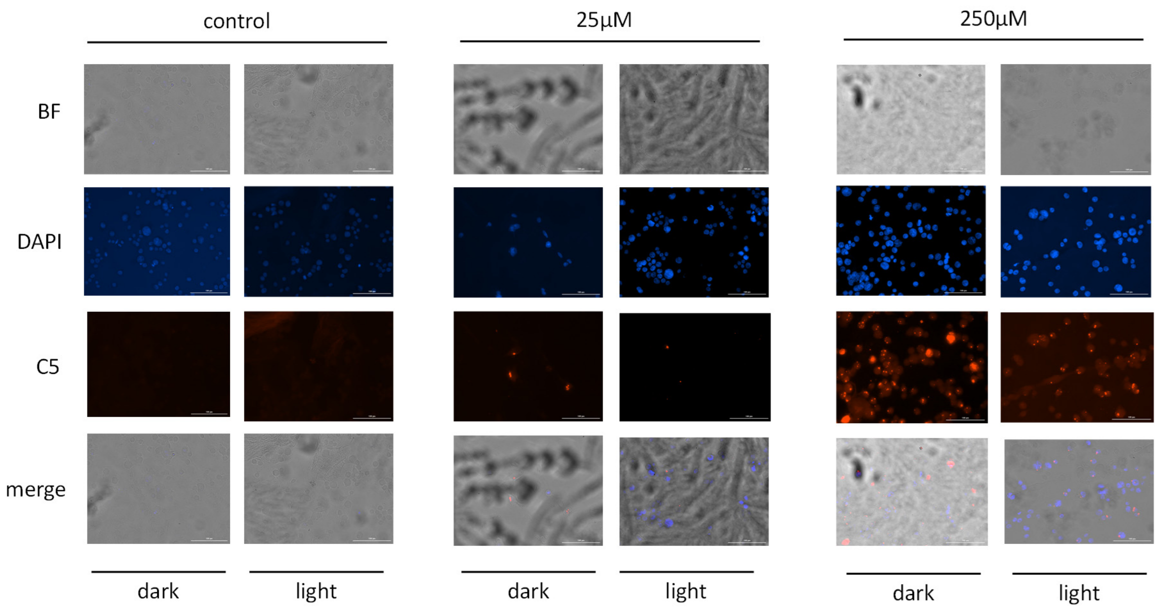

Supplementary Figure S4: Porphyrins are efficiently delivered by EVs to CRC cells in a dose-dependant manner. Detected using fluorescence, counter-stained with DAPI. Significantly more C5 is delivered, and to significantly more cells, when 250µL of C5 is used for EV loading.

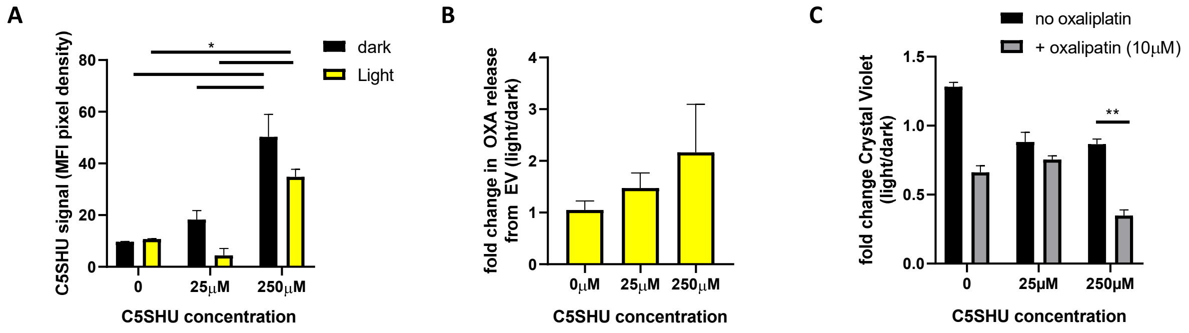

Supplementary Figure S5: C5SHU and light influence release and delivery from EVs. Quantification of C5SHU delivered to SW480 cells was performed (A) by measurement of the mean fluorescence intensity (MFI) values (see supplementary figure 2). Light exposure did not significantly increase the release of oxaliplatin from EVs co-loaded with C5SHU, although there was a trend towards increased release with increasing C5SHU concentration (B). Light exposure significantly decreased the viability of cells treated with Oxa-C5-EVs loaded with 250µM C5SHU (C). * = $p < 0.05$, ** = $p < 0.01$.

Supplementary references:

[S1] Silva S, Pereira PMR, Silva P, Paz FAA, Faustino MAF, Cavaleiro JAS, et al. Porphyrin and phthalocyanine glycodendritic conjugates: synthesis, photophysical and photochemical properties. Chemical communications, 2012;48(30):3608-3610.

[S2] Bhaumik J, Weissleder R, McCarthy JR. Synthesis and Photophysical Properties of Sulfonamidophenyl Porphyrins as Models for Activatable Photosensitizers. J Org Chem 2009;74(16):5894-5901.
